# Contactless and robust dielectric microspheres-assisted surface-enhanced Raman scattering sensitivity improvement for anthrax biomarker detection

**DOI:** 10.3389/fchem.2022.1057241

**Published:** 2022-11-15

**Authors:** Mengyi Ge, Wenfeng Zhao, Yue Han, Hongwei Gai, Chenghua Zong

**Affiliations:** School of Chemistry and Materials Science, Jiangsu Normal University, Xuzhou, Jiangsu, China

**Keywords:** surface-enhanced Raman scattering, dielectric microsphere, detection, dipicolinic acid, polydimethylsiloxane (PDMS)

## Abstract

This report presents a contactless and robust dielectric microspheres (DMs)-assisted surface enhanced Raman scattering (SERS) enhancement method to improve SERS detection sensitivity detection sensitivity. DMs that could focus and collect light were embedded within the polydimethylsiloxane (PDMS) film to avoid direct contact with the analytical solution and improve detection reliability. The as prepared DMs embedded PDMS (DMs-PDMS) film was integrated with a microfluidic technique to enhance the SERS signal of a liquid substrate. Detection in microfluidic systems can reduce reagent consumption, shorten assay time, and avoid evaporation of the colloid substrate solution. The robustness and potential influencing factors of DMs-PDMS film assisted SERS enhancement (DERS) were evaluated using 4-aminothiophenol (4-ATP) as the Raman probe. The sensing performance of the proposed method toward dipicolinic acid (DPA) was evaluated, and an evident signal intensification was obtained. Remarkably, the DMs-PDMS film can also be implemented on solid substrates. A proof-of-concept experiment was performed by covering the DMs-PDMS film directly over an AgNPs@Si solid substrate wherein a 5.7-fold sensitivity improvement was achieved.

## Introduction

Raman spectroscopy, particularly surface-enhanced Raman scattering (SERS), is suitable for sensing applications because it can provide molecular fingerprint information that uniquely identifies target analytes from a complex matrix (Bell et al., 2005; Cheng et al., 2009; Naqvi et al., 2021). Moreover, it is time-saving and can be completed within a few minutes, and it offers high sensitivity. It is well known that SERS signals are highly dependent on the substrate. Substrates with diverse structures and compositions have been designed and synthesized. These substrates provide excellent SERS enhancement and contribute significantly to the application of SERS. Nevertheless, sophisticated synthesis routes are unsuitable for large-scale production and lack universality. Therefore, developing a facile method that can indiscriminately enhance the SERS signal, regardless of the substrate, is of great significance.

Dielectric microspheres (DMs), which can focus and collect light, provide an alternative approach to optical signal enhancement (Zhang Q. Q. et al., 2021). Schwarte et al. reported that TiO_2_ colloids can act as lenses to enhance the fluorescence of single molecules (Schwartz et al., 2010). Yang et al. demonstrated the use of three and 9.75 μm DMs as *in situ* lenses for fluorescence signal enhancement (Yang and Gijs 2013). Compared with fluorescence, the application of DMs in Raman spectroscopy is relatively undeveloped. DMs were first exploited for Raman enhancement in 2007 and have been optimized since then (Yi et al., 2007), (Dantham et al., 2011; Du et al., 2011). In 2015, Yi et al. reported enhanced confocal Raman detection using polystyrene (PS) microsphere array. The maximum enhancement ratio of Raman intensity was up to 14.6 using 4.94-μm diameter PS microspheres (Yan et al., 2015). These results promote the research interest in coupling of DMs to metallic SERS substrates to further boost SERS sensitivity (Lu et al., 2009; Le Beulze et al., 2017; Nam et al., 2019; Hong et al., 2020). For example, Wang et al. deposited a silver layer onto a DMs surface using electron beam evaporation. The Raman signal of rhodamine 6G molecules measured in presence of DMs was up to seven times larger than that measured using a flat silver film alone (Wang et al., 2019). A uniform raspberry-like nanostructure composed of noble metal nanoparticles (Au_n_ or Ag_n_) and silica particles (80 or 100 nm) was synthesized by taking the advantage of well-studied electrostatic attractions (Hong et al., 2022). Recently, a sandwich structure was designed by immobilizing polymethyl methacrylate (PMMA) microspheres, self-assembling silver nanoparticles (AgNPs), and depositing SiO_2_ microspheres. In this case, the PMMA and SiO_2_ microspheres constituted the dielectric microenvironment that enriched the light illumination at AgNPs for SERS detection (Hou et al., 2022). In practice, DMs with larger sizes are usually expected to be duplicated and compatible with various types of instruments, such as portable Raman spectrometers. However, the currently used DMs typically have diameters of <10 μm. Although they show excellent SERS signal enhancement, their enhancement efficiency is highly dependent on their size and assembly structure, which is a significant challenge for accurate and reliable SERS detection. Moreover, the most commonly used strategy of directly assembling DMs and metallic NPs lacks universality, and the DMs cannot be reused.

Dipicolinic acid (DPA), one of the main ingredients of anthrax spores (accounts for 5–15% of the dry mass of spores), has been adopted as a significant biomarker for the detection of anthrax (Yin and Tong 2021). Various DPA detection methods have been reported over the past decade. Liquid chromatography and gas chromatography/ion mobility mass spectrometry are two common laboratory-based DPA detection techniques (Watabe et al., 1988; Snyder et al., 1999). In addition to the lengthy analysis time, these methods suffer from low sensitivity (Paulus 1981; Dworzanski et al., 1997). Recently, colorimetric and fluorescent methods have attracted increasing attention owing to their simplicity and visibility (Yang et al., 2020; Koo et al., 2021). Complexing DPA with Tb^3+^ or Eu^3+^ is one of the most commonly used strategies (Yilmaz et al., 2010; Na et al., 2020). Despite significant improvements, these methods are susceptible to interference from other molecules either by binding directly to the probe or by binding competitively with DPA.

In this study, a flexible and robust DMs embedded polydimethylsiloxane (DMs-PDMS) film was fabricated and used as a versatile and universal Raman scattering enhancer to improve the sensitivity of SERS detection. The DMs that could enrich the SERS signals were insulated from the solution, ensuring detection reliability and repeatability. The excellent optical transparency of PDMS guarantees an efficient passing through of light for SERS excitation and collection. Combined with microfluidic technology, the as-prepared DMs-PDMS film can be used as a universal platform to enhance the SERS signals of the liquid substrate. We attempted to use this DMs-PDMS film-assist SERS enhancement approach (DERS) to detect DPA, and a significant signal improvement was achieved. Moreover, by simply covering the DMs-PDMS film over a solid substrate, it is encouraging to find that the SERS signals of the solid substrate can also be enhanced.

## Materials and methods

### Reagents and instruments

Na_3_C_6_H_5_O_7_.2H_2_O, AR, was purchased from Alfa Aesar, silver nitrate (AgNO_3_), Dipicolinic acid (DPA) and 4-aminothiophenol (4-ATP) were from Aladdin (China); 3-Aminopropyltriethoxysilane (APTES) was from J&K Scientific ltd. Sylgard 184 PDMS oligomer and curing agent were from Dow Corning, High refractive index barium titanate solid glass microspheres (BTGMs) was from Cospheric (US). H94-25c 4 “single lithography machine was purchased from Sichuan Nanguang Vacuum Technology Co., Ltd. Plasmon cleaner (PDC-32G-2*) was provided by Harrick Plasma (US). Raman spectrometer BWS415-785S was from B&WTEK (US).

### Fabrication of the DMs-PDMS film

**SCHEME 1:**
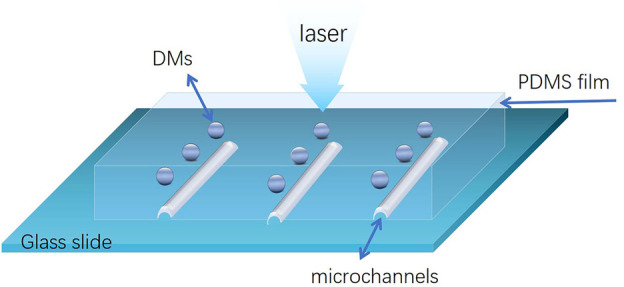
Schematic representation of the DMs-PDMS film setup.

The DMs-PDMS films with microchannels was fabricated following a modified photolithography process simply described as follows: A positive photoresist mold was firstly fabricated according to a previous report (Chen et al., 2020). Then PDMS and initiators were mixed at a volume ratio of 10:1, and degassed by vacuum, after that the PDMS precursor was gently poured on the positive photoresist mold until the mold was immersed. DMs were embedded in the PDMS precursor at the top of photoresist patterns. The obtained mold with PDMS precursor was heated at 85 C for 25°min. Later after, the PDMS piece with patterns was peeled off from the mold, punctured reservoirs and bonded to clean glass substrate by oxygen plasma. Finally, the DMs-PDMS film with microchannels was formed (Scheme 1). Notably, microchannels with different width and depth were fabricated through the same procedures but using different photoresist molds. For evaluating the influence of refractive index (RI), two kinds of DMs (BTGMs and common glass microbead) were embedded at different areas of the same PDMS film. For using in solid SERS substrate, the DMs was embedded in a PDMS film directly without using photoresist mold.

### Preparation of the silver nanoparticles and investigation of the DERS effect

Silver nanoparticles (AgNPs) used as the SERS substrate were synthesis by using a method reported previously (Qu et al., 2016). In brief, 36 mg AgNO_3_ was dissolved in 100 ml DI water and heated rapidly to boil under vigorous stirring. Then the freshly prepared trisodium citrate solution (1%, 4 ml) was added into the boiling solution, resulting a rapidly brown to gray color changes within a few minutes. The solution was boiled for another 30 min to ensure the complete reduction of silver nitrate, and then AgNPs solution was obtained. The as-prepared AgNPs were concentrated for 10-fold and then stored in dark for use.

For testing the DERS effect, 4-ATP, a commonly used SERS probe was used. 4 μl 4-ATP (2 μM) solution was mixed with 35 μl AgNPs and incubated for 5 min. The mixture was filled into the microchannels. Then SERS signals in the presence and absence of DMs was collected and compared. Similar procedures were also employed for investigating the influence factors and robustness of the DERS effect.

Proof-of-concept experiments for testing the enhancing effect of the DMs-PDMS film on a solid substrate was carried out as follows: First, silicon slide was cleaned sequential ultrasonication in acetone, ethanol and deionized water for 10 min and then treated with H_2_SO_4_/H_2_O_2_ (3:1 (v/v) H_2_SO_4_ (98%)/H_2_O_2_ (30%) at 100°C for 30 min to derive a hydroxyl surface. Second, after washing, the silicon slide was immersed into a 6% solution of APTES in ethanol for 1 h followed by a profusely rinsing. Third, the silane modified silicon slide was submerged into AgNPs for 3 h, then an AgNPs coated silicon slide was obtained (AgNPs@Si). After dry in nitrogen, the AgNPs@Si substrate was immersed into 4-ATP solution in ethanol, and washer by DI water 1 h later. After that, the DMs-PDMS film was placed on the AgNPs@Si substrate and SERS spectra were collected.

### Dipicolinic acid detection evaluation

To evaluate the influence of HNO_3_ on sensitive detection of DPA, aqueous solution of nitric acid with different concentrations was prepared. Then DPA solutions (0.02 mM) were prepared by using these nitric acid solutions. 4 μl of this DPA solution was mixed with 35 μl AgNPs and incubated for 5 min (the final concentrations of HNO_3_ is 100, 20, 10, 2, 1, 0.2 (mM), respectively). After that the mixture was filled into the microchannels and the SERS spectra were collected.

For quantitative measurements, standard solutions of DPA with different concentrations were prepared at the optimal HNO_3_ concentration. 4 μl of the DPA solution was mixed with 35 μl AgNPs to a final concentration of 100 μM, 50 μM, 10 μM, 1 μM and 0.1 μM, respectively. After incubated for 5 min, the mixture was added into the microchannels and the SERS spectra was collected.

## Results and discussion

### DERS effect investigation of the DMs-PDMS film

**FIGURE 1:**
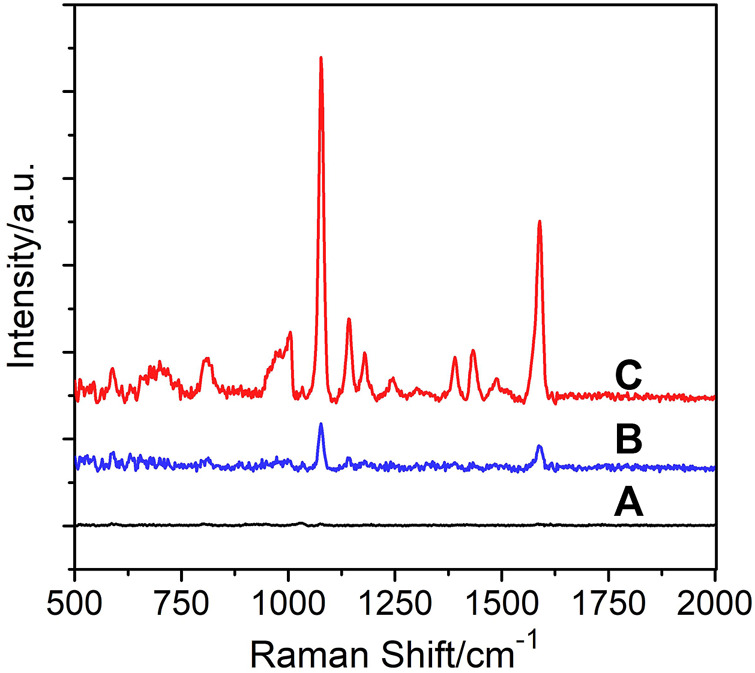
SERS signal of 0.2 μM 4-ATP obtained **(A)** in the absence of AgNPs and DMs; **(B)** in the presence of AgNPs but the absence of DMs; **(C)** in the presence of both AgNPs and DMs.

To test the DERS effect of the as-prepared DMs-PDMS film, AgNPs, one of the most commonly used SERS substrates, were synthesized according to a previous study (Qu et al., 2016). 4-ATP, which can absorb AgNPs *via* chemical coordination, was chosen as the Raman probe. As shown in Figure 1, no apparent Raman signal was observed when 0.2 μM 4-ATP solution alone was excited (A); however, in the presence of AgNPs, enhanced Raman signals can be obtained (B); and this signal can be further enhanced in the presence of DMs (C), revealing an obvious DERS effect. The DERS effect might be attributed to the optical focusing and collecting properties of DMs. We proposed that the DMs in the PDMS film can act as lens to focus both the exciting light and the Raman scattering light, thus improving the excitation and collecting efficiency and providing an enhanced SERS intensity.

**FIGURE 2:**
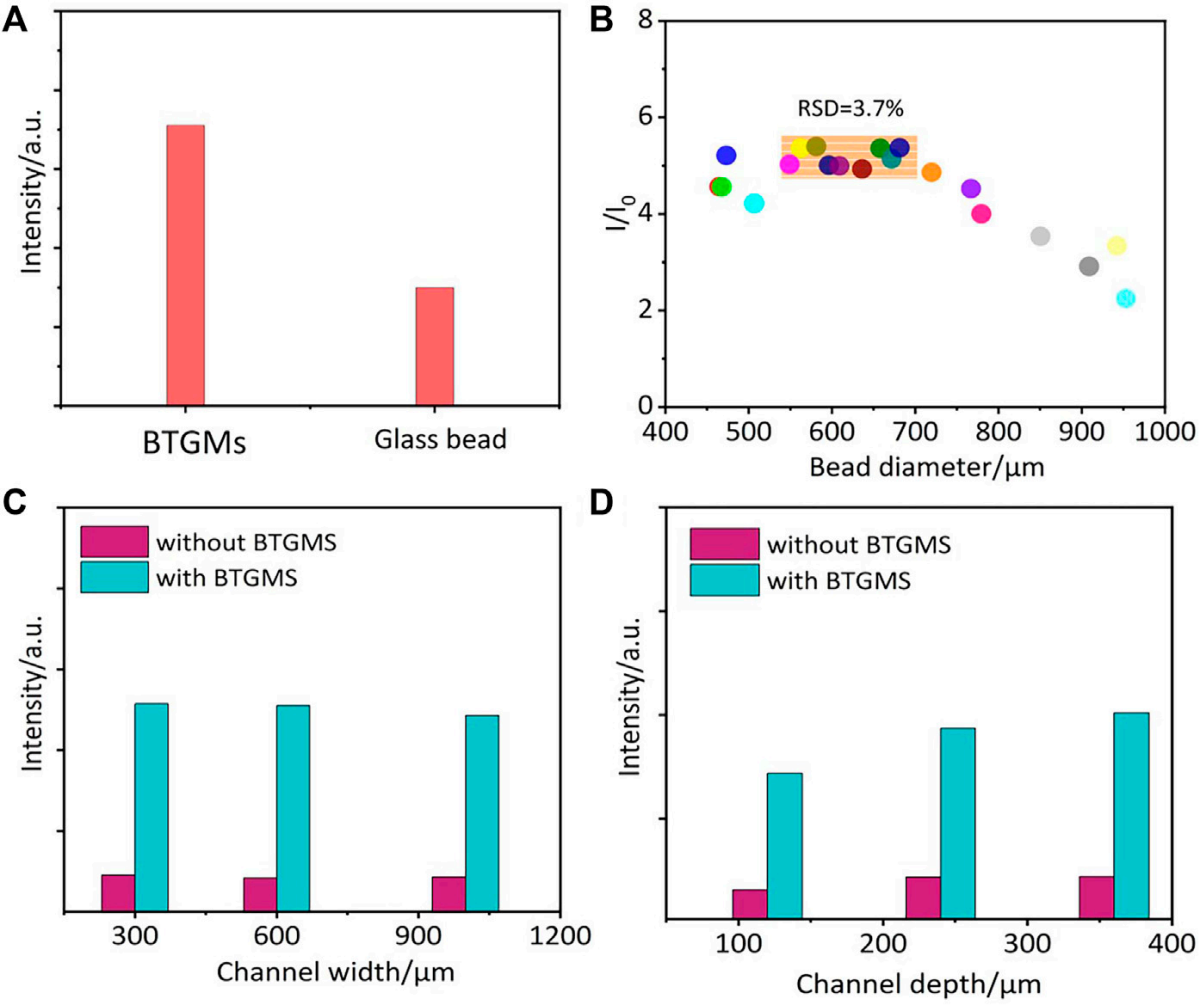
The influence of the bead RI **(A)** and diameter **(B)** on the DERS effect. Comparable SERS signals of 4-ATP that obtained in microchannels with different widths **(C)** and depths **(D)**. I and I_0_ are the peak intensity of 4 ATP (1077cm^−1^) collected in the presence and absence of BTGMS, respectively.

Optical focusing and collecting ability of DMs may be related to their refractive index (RI). Two types of DMs with different RI (common glass microbeads with an RI of approximately 1.5 and BTGMs with an RI of approximately 1.9) were embedded in different areas of the same PMDS film. As shown in Figure 2A, under the same conditions, the SERS signals obtained from the BTGMs were much higher than those obtained from the common glass microbeads. Therefore, BTGMs were used in the following experiments. Notably, a similar RI-dependent signal increase has been observed in previous reports on fluorescence (Schwartz et al., 2010; Zhang Q. et al., 2021).

### Robustness of the DERS effect

To test the robustness of the DERS effect, BTGMs of different sizes were embedded into the PDMS film, and the size influence on the enhancing efficiency was investigated. As shown in Figure 2B, the DERS effect was observed in all used BTGMs. The highest signals were obtained in the size range (550–680) ± 20 μm (Figure 2B). In particular, the DERS effect was quite stable in this size range (RSD <3.7%); thus, a slight fluctuation in the DM size would not induce significant SERS signal changes. Further increasing the size beyond this range led to a decrease in the enhancing efficiency, which might result from the reduced microsphere transmittance. To test whether the width and depth of the microfluidic channels in PDMS affect the DERS effect, DMs-PDMS films with microchannels of different widths (300, 600 and 1,000 μm) and depths (120, 240 and 360 μm) were fabricated. As shown in Figures 2C,D, in all the detected samples, the SERS signals obtained in the presence of BTGMs were much higher than those in the absence of BTGMs, further confirming the ability of the BTGM-PDMS film in SERS signal enhancement. As shown in Figure 2C, the channel width did not affect the DERS effect (Figure 2C). For microchannels with different depths, stable enhancement was obtained as the channel depth increased from 240 to 360 μm (Figure 2D). These results confirmed the robustness of the DERS effect. Notably, DMs of 650 μm, and microchannels with width and depth of 600 and 360 μm, respectively, were used in the following experiments.

### Sensing performance evaluation

**FIGURE 3:**
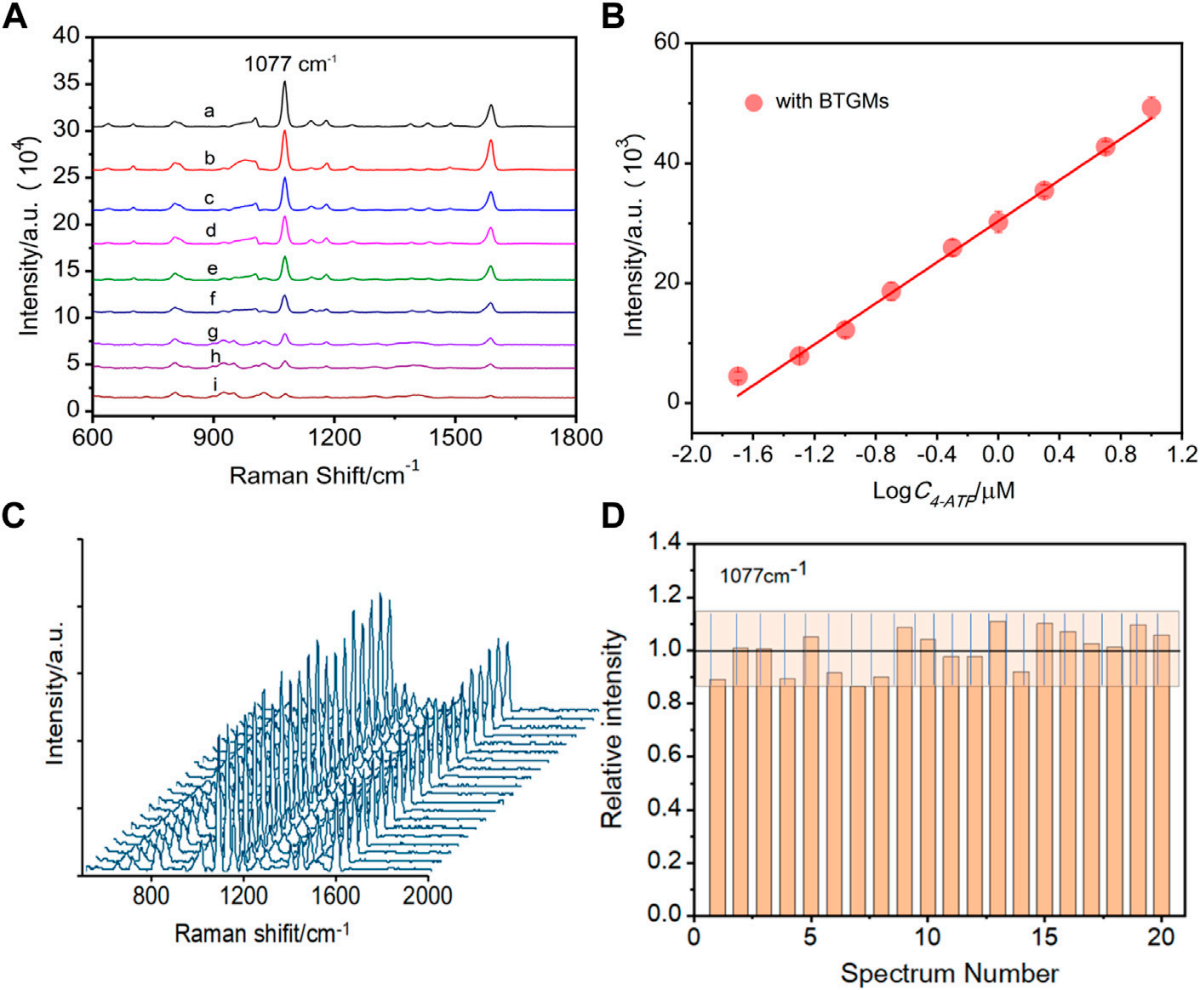
SERS spectra of different concentrations of 4-ATP obtained in the presence of BTGMs **(A)** (a–i: 10 μM, 5 μM, 2 μM, 1 μM, 0.5 μM, 0.2 μM, 0.1 μM, 50 nM and 20 nM, respectively). **(B)** Linear relationship between the intensity at 1,077 cm^−1^ of 4-ATP with its concentration. **(C)** SERS spectra of 4-ATP were acquired from 20 randomsiteswith the DERS effect (in the presence of the BTGMs). **(D)** Corresponding bar chart for the peak intensity at 1,077 cm^−1^, the grating zone is indicated with ±13% intensity variation. DMs of 650 μm and microchannel with a width and depth of 600 and 360 μm, respectively, were used.

A linear relationship is crucial for quantitative analysis. To explore the influence of DMs on the linear relationship, the SERS signals of different concentrations of 4-ATP (20 nM–10 μM) in the presence of DMs (BTGMs) were collected. As demonstrated in Figures 3A,B good linear relationship was obtained, indicating that the linear relationship was not weakened by the BTGMs, which laid the basis for subsequent detection. We envision that the robustness of the DMs-PDMS film should lead to repeatable SERS enhancement. To test this, we recorded the SERS spectra of 4-ATP (1 μM) from 20 randomly selected spots with BTGMs (Figure 3C) and quantitatively presented the intensity variation of the characteristic peak at 1,077 cm^−1^ (Figure 3D). The obtained SERS signals exhibit good repeatability with a relative standard deviation (RSD) of 13%.

### Applications in dipicolinic acid analysis

**FIGURE 4:**
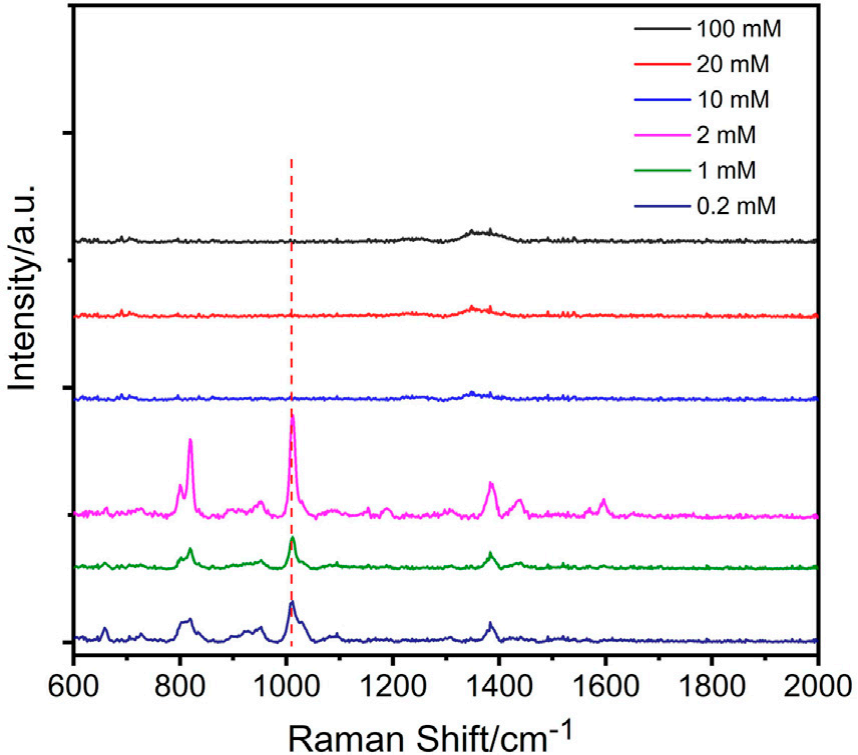
Influence of HNO_3_ on the detection of DPA (the concentration of DPA is 20 μM).

Encouraged by the above results, we explored the possibility of using the proposed DERS effect for DPA detection. It has been reported that acidic conditions are favored for SERS analysis of DPA (Cowcher et al., 2013). Figure 4 demonstrates the relative SERS intensities for comparable DPA concentrations obtained in the presence of different concentrations of HNO_3_. Most prominent SERS signals were observed at an HNO_3_ concentration of 2 mM. Further increasing the HNO_3_ concentration to 10 mM led to a significant decrease in the signal, which may be attributed to the high acidity-induced dissolution of the AgNPs.

**FIGURE 5:**
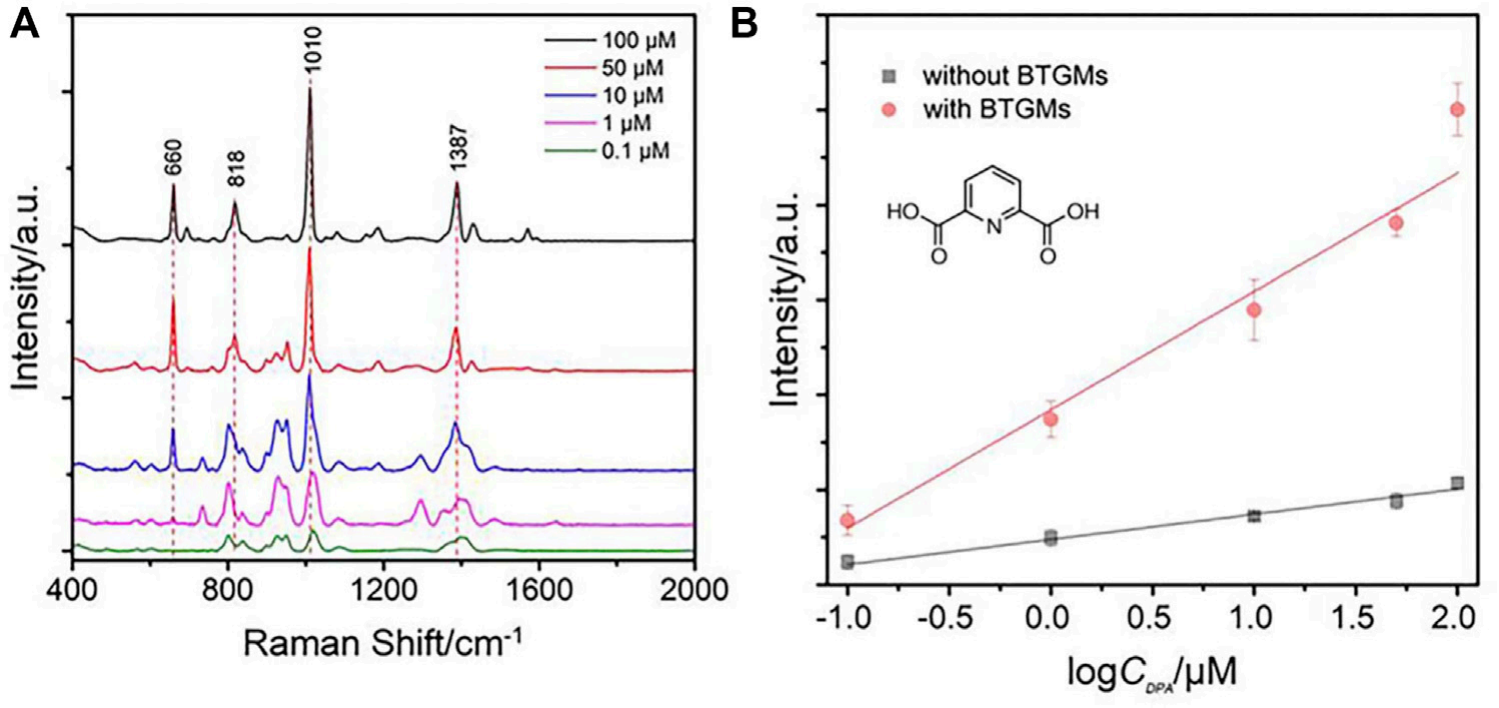
SERS spectra of DPA with different concentrations **(A)** (100 μM, 50 μM, 10 μM, 1 μM, and 0.1 μM). **(B)** Comparable linear relationship between the intensity at 1,010 cm^−1^ of DPA with its concentration. The chemical structure of DPA is shown.

For quantitative measurement, different concentrations of DPA solution in 2 mM HNO_3_ were mixed with the AgNPs. The mixture was then filled into the microchannels of the BTGM-PDMS film, and the corresponding SERS signals were collected. Figure 5A shows the superimposed fingerprints from which the characteristic peaks corresponding to DPA can be identified (660 cm^−1^, 818 cm^−1^, 1,010 cm^−1^, and 1,387 cm^−1^ peaks attributed to C = C ring bending vibration, C-H in-plane bending vibration, symmetric pyridine ring stretching vibration, and O-C-O symmetric bending vibration, respectively (Cheng et al., 2009). Quantitative data of the SERS intensity at 1,010 cm^−1^ were extracted from these spectra and further correlated with the DPA concentrations. As shown in Figure 5B, the SERS signals increased with increasing DPA in both the cases. In particular, the slope of the curve obtained in the presence of BTGMs was 5 times larger than that of the original curve without BTGMs, revealing that the sensitivity for DPA detection was increased by approximately 5 times by the DERS effect (the noise from the detection instrument was not affected by the DERS effect). Based on a signal-to-noise ratio of 3, the limit of detection (LOD) for DPA was calculated to be 15 nM and that was lower than other AgNP-based SERS methods for DPA detection (Table 1).

**TABLE 1:** Comparison of different methods for DPA detection.

Method	Sensing platform	Detection limit	Ref
Fluorescent	Lanthanide-based surface receptor	25 nM	Yilmaz et al. (2010)
Fluorescent	Hetero MOF	0.248 μM	Shen et al. (2020)
Fluorescent/colorimetric	Eu^3+^-modified AuNPs	0.31 μM for colorimetric and 17 nM for fluorescent assay	Yin and Tong (2021)
colorimetric	Gold nanoparticles	2 μM	Baig and Chen (2018)
colorimetric	Upconversion nanoparticles	0.9 μM	Cheng et al. (2019)
SERS	AgNPs	20 ng/20 μL	Bell et al. (2005)
SERS	AgNPs	29.9 nM	Cowcher et al. (2013)
SERS	AgNPs	15 nM	This work

### Additional applications

**FIGURE 6:**
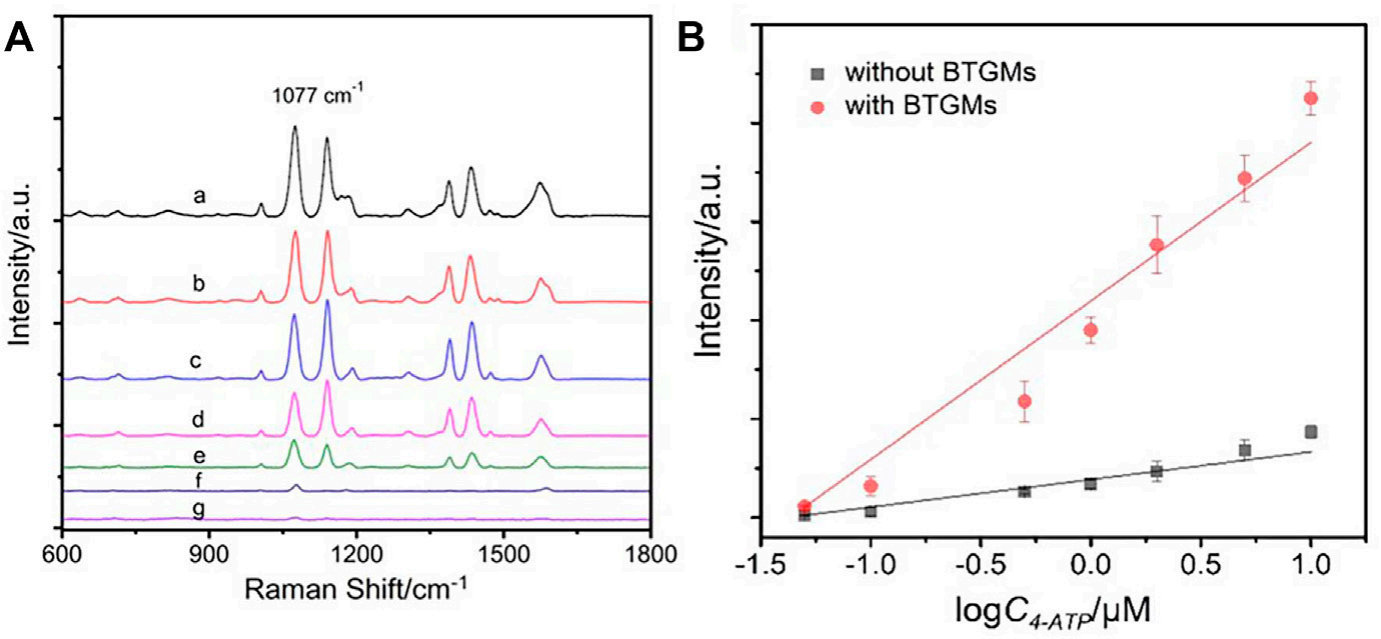
SERS spectra of different concentrations of 4-ATP obtained from the AgNPs@Si substrate covered by the BTGMs-PDMS film **(A)** (a–g: 10 μM, 5 μM, 2 μM, 1 μM, 0.5 μM, 0.1 μM and 0.05 μM, respectively). **(B)** Linear relationship for SERS detection of 4-ATP obtained in the presence and absence of the BTGMS-PDMS film, respectively.

Inspired by the initial results for enhancing the SERS signals of the AgNP colloidal substrate, we explored the possibility of using the as-prepared BTGM-PDMS film for enhancing solid SERS substrates. To prepare the solid substrate, a silicon slide was silanized with APTES. The resulting amino-terminated silane monolayer on the silicon surface was used to react with the AgNPs, and a solid AgNPs@Si substrate was formed after drying in nitrogen. The as-formed solid substrate was incubated with different concentrations of the Raman probe (4-ATP). The corresponding SERS spectra were collected directly by covering the BTGM-PDMS film over the AgNPs@Si substrate. PDMS exhibits high adhesion and mechanical flexibility; thus, it can firmly adhere to the AgNPs@Si substrate. As shown in Figure 6, an excellent linear relationship was obtained. Notably, the slope of the enhanced curve was 5.7 times larger than that of the original curve without BTGMs, revealing the capability of the BTGM-PDMS film as a versatile SERS enhancer.

## Conclusion

In conclusion, we report a contactless and DMs-assisted sensitivity improvement method for SERS detection. The method takes advantage of the excellent properties of PDMS (high transparency, high adhesiveness, and mechanical flexibility) and high optical focusing and collecting ability of DMs to provide improved sensitivity for the rapid and sensitive detection of analytes. By using 4-ATP as a probe molecular, the robustness and sensing performance of the DERS effect has been evaluated. By taking advantage of this DERS effect, the sensitivity for SERS detection of DPA was improved by 5-fold. Meanwhile, the as-prepared BTGM-PDMS film can be extended to enhance the SERS signals of solid substrates. A proof-of-concept experiment was performed using an AgNPs@Si substrate, and a 5.7-fold sensitivity improvement was achieved. The proposed strategy is expected to open new avenues for the design of ultrasensitive SERS assays.

## Data Availability

The original contributions presented in the study are included in the article/supplementary material, further inquiries can be directed to the corresponding author.
