# Corrigendum: Contactless and robust dielectric microspheres-assisted surface-enhanced Raman scattering sensitivity improvement for anthrax biomarker detection

**DOI:** 10.3389/fchem.2023.1161985

**Published:** 2023-03-09

**Authors:** Mengyi Ge, Wenfeng Zhao, Yue Han, Hongwei Gai, Chenghua Zong

**Affiliations:** School of Chemistry and Materials Science, Jiangsu Normal University, Xuzhou, Jiangsu Province, China

**Keywords:** surface-enhanced Raman scattering, dielectric microsphere, detection, dipicolinic acid, polydimethylsiloxane (PDMS)

In the original article, there was a spelling mistake in the name of the second author. The correct name appears above.

In the original article, there were errors in Table 1, page 7, as published. Three reference links and their corresponding references were missing; “Eu^3−^” should have been “Eu^3+^”; “0.248 p.m.” should have been “0.248 μM”; “0.13 p1\4” should have been “0.31 μM”; “0.9 [tM” should have been “0.9 μM”; “20 n9.120 pL” should have been “20 ng/20 μL”; the reference Baiv et al. (2018) should have been Baig and Chen (2018) and Cheng et al. (2009) should have been Cheng et al. (2019). There was also a typo in the caption. The corrected Table 1 and its caption, as well as the additional references, appear below.

**TABLE 1:** Comparison of different methods for DPA detection.

Method	Sensing platform	Detection limit	Ref
Fluorescent	Lanthanide-based surface receptor	25 nM	Yilmaz et al. (2010)
Fluorescent	Hetero MOF	0.248 μM	Shen et al. (2020)
Fluorescent/colorimetric	Eu^3+^-modified AuNPs	0.31 μM for colorimetric and 17 nM for fluorescent assay	Yin and Tong (2021)
Colorimetric	Gold nanoparticles	2 μM	Baig and Chen (2018)
Colorimetric	Upconversion nanoparticles	0.9 μM	Cheng et al. (2019)
SERS	AgNPs	20 ng/20 μL	Bell et al. (2005)
SERS	AgNPs	29.9 nM	Cowcher et al. (2013)
SERS	AgNPs	15 nM	This work

In the original article, the abbreviation of DMs-PDMS was wrongly modified during production. Corrections have been made to the **abstract**, page 1. The third sentence previously stated:

“The as prepared DMs embedded PDMS DMs PD MS film was integrated with a microfluidic technique to enhance the SERS signal of a liquid substrate.”

The corrected sentence is as follows:

“The as prepared DMs embedded PDMS (DMs-PDMS) film was integrated with a microfluidic technique to enhance the SERS signal of a liquid substrate.”

Additionally, all instances of the abbreviation “DMs PDMS” have been corrected to “DMs-PDMS” throughout the **abstract**, page 1; **Introduction,** page 2, last paragraph; **Material and methods** sub-heading, “*Fabrication of the DMs PDMS film*”, page 2; as well as the **Results and discussion** subheading, “*DERS effect investigation of the DMs PDMS film*”, page 3.

In the original article, there was an error in Figure 3 as published. For the repeatability study, the SERS spectra and the relative standard deviation value were obtained from 20 random spots, as shown in Figure 3C, not 15. The corrected Figure 3 and its caption appear below.

**FIGURE 3:**
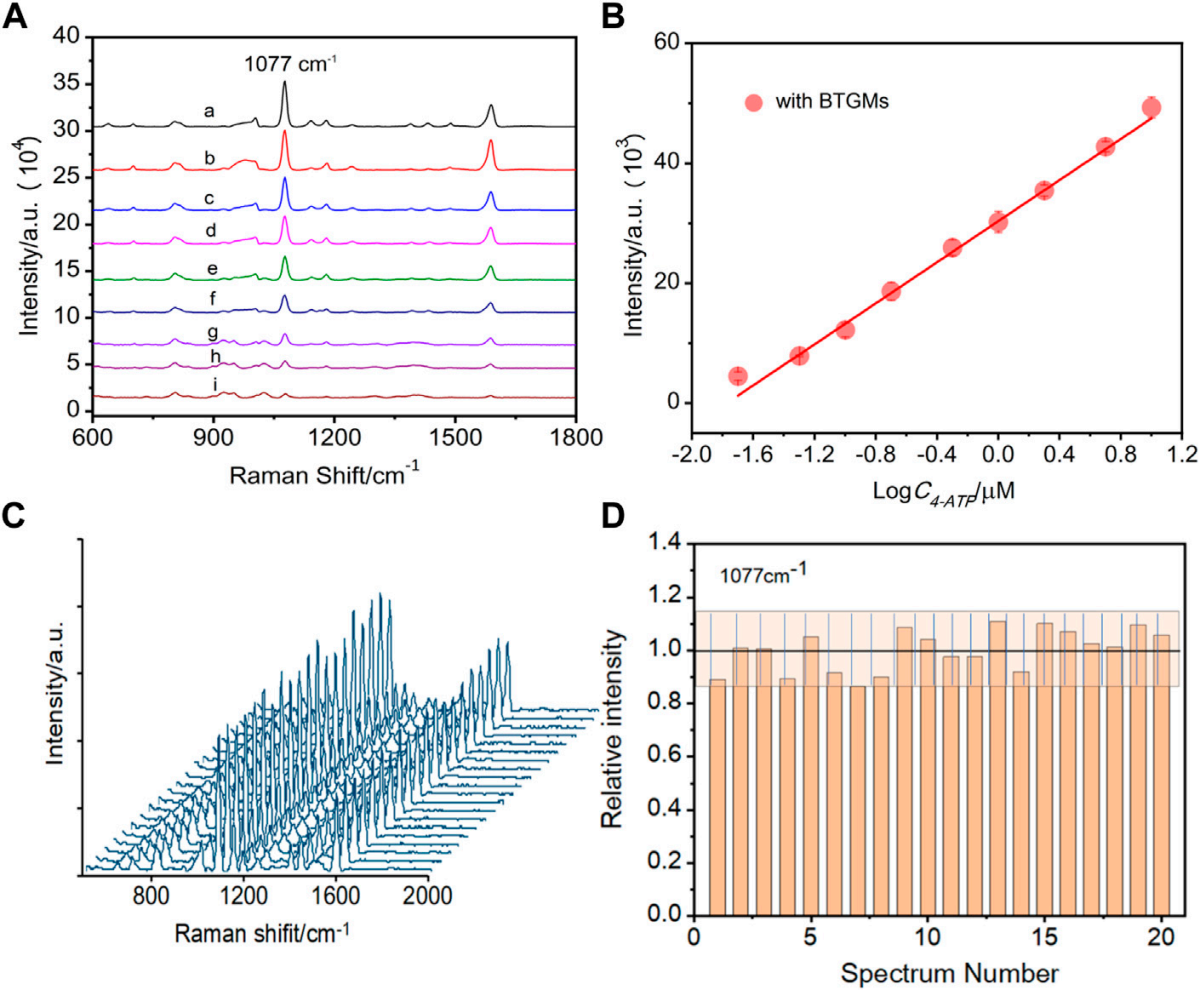
SERS spectra of different concentrations of 4-ATP obtained in the presence of BTGMs **(A)** (a–i: 10 μM, 5 μM, 2 μM, 1 μM, 0.5 μM, 0.2 μM, 0.1 μM, 50 nM and 20 nM, respectively). **(B)** Linear relationship between the intensity at 1,077 cm^−1^ of 4-ATP with its concentration. **(C)** SERS spectra of 4-ATP were acquired from 20 random sites with the DERS effect (in the presence of the BTGMs). **(D)** Corresponding bar chart for the peak intensity at 1,077 cm^−1^, the grating zone is indicated with ±13% intensity variation. DMs of 650 μm and microchannel with a width and depth of 600 and 360 μm, respectively, were used.

Consequently, in **Results and discussion**, “*Sensing performance evaluation*”, page 4, “15 randomly selected spots” should be “20 randomly selected spots”.

The authors apologize for these errors and state that this does not change the scientific conclusions of the article in any way. The original article has been updated.
